# Microbial Community Shifts Reflect Losses of Native Soil Carbon with Pyrogenic and Fresh Organic Matter Additions and Are Greatest in Low-Carbon Soils

**DOI:** 10.1128/AEM.02555-20

**Published:** 2021-03-26

**Authors:** Thea Whitman, Silene DeCiucies, Kelly Hanley, Akio Enders, Jamie Woolet, Johannes Lehmann

**Affiliations:** a Department of Soil Science, University of Wisconsin—Madison, Madison, Wisconsin, USA; b Soil and Crop Sciences, School of Integrative Plant Science, Cornell University, Ithaca, New York, USA; c Atkinson Center for a Sustainable Future, Cornell University, Ithaca, New York, USA; University of California, Davis

**Keywords:** ^13^C, NEON, biochar, decomposition, mineralization, positive priming, soil, stable isotopes

## Abstract

Soil organic carbon (SOC) plays an important role in regulating global climate change, carbon and nutrient cycling in soils, and soil moisture. Organic matter (OM) additions to soils can affect the rate at which SOC is mineralized by microbes, with potentially important effects on SOC stocks. Understanding how pyrogenic organic matter (PyOM) affects the cycling of native SOC (nSOC) and the soil microbes responsible for these effects is important for fire-affected ecosystems as well as for biochar-amended systems. We used an incubation trial with five different soils from National Ecological Observatory Network sites across the United States and ^13^C-labeled 350°C corn stover PyOM and fresh corn stover OM to trace nSOC-derived CO_2_ emissions with and without PyOM and OM amendments. We used high-throughput sequencing of rRNA genes to characterize bacterial, archaeal, and fungal communities and their responses to PyOM and OM in soils that were previously stored at −80°C. We found that the effects of amendments on nSOC-derived CO_2_ reflected the unamended soil C status, where relative increases in C mineralization were greatest in low-C soils. OM additions produced much greater effects on nSOC-CO_2_ emissions than PyOM additions. Furthermore, the magnitude of the microbial community composition change mirrored the magnitude of increases in nSOC-CO_2_, indicating that a specific subset of microbes was likely responsible for the observed changes in nSOC mineralization. However, PyOM responders differed across soils and did not necessarily reflect a common “charosphere.” Overall, this study suggests that soils that already have low SOC may be particularly vulnerable to short-term increases in SOC loss with OM or PyOM additions.

**IMPORTANCE** Soil organic matter (SOM) has an important role in global climate change, carbon and nutrient cycling in soils, and soil moisture dynamics. Understanding the processes that affect SOM stocks is important for managing these functions. Recently, understanding how fire-affected organic matter (or “pyrogenic” organic matter [PyOM]) affects existing SOM stocks has become increasingly important, due to both changing fire regimes and interest in “biochar,” pyrogenic organic matter that is produced intentionally for carbon management or as an agricultural soil amendment. We found that soils with less SOM were more prone to increased losses with PyOM (and fresh organic matter) additions and that soil microbial communities changed more in soils that also had greater SOM losses with PyOM additions. This suggests that soils that already have low SOM content may be particularly vulnerable to short-term increases in SOM loss and that a subset of the soil microbial community is likely responsible for these effects.

## Abstract (precis)

Soil organic matter (SOM) has an important role in global climate change, carbon and nutrient cycling in soils, and soil moisture dynamics. Understanding the processes that affect SOM stocks is important for managing these functions.

## INTRODUCTION

Soil organic matter (SOM) supports a wealth of benefits in soil systems. This includes providing organic nutrients, binding toxic compounds, increasing the soil water-holding capacity, and storing soil organic carbon (SOC). Globally, soils hold large stocks of carbon (C), twice the amount of C held in living biomass or in the atmosphere (1). Understanding the processes that control the stocks and fluxes of C in and out of the soils is thus essential for mitigating climate change as well as for sustainable agricultural management (2). Recently, the importance of understanding the role of fire-affected organic matter (or pyrogenic organic matter [PyOM], *sensu* Zimmerman and Mitra [3]) in contributing to SOC stocks has become increasingly salient. PyOM can make up large portions of soil carbon stocks, particularly in fire-affected ecosystems (4), where it can represent over 60% of the total SOC (5). Its persistence in soils has led to interest in its role in offsetting the climate impacts of natural wildfires (4) as well as the possibility of its intentional production for the stabilization of organic matter (OM), in which case it is often referred to as “biochar” (6, 7). However, in order to quantify its net effect on C stocks and fluxes, it is essential to understand not only the persistence of pyrogenic C (PyC) itself but also its effect on the native SOC (nSOC) present before PyC additions.

After interest was sparked in the potential of PyC for climate change mitigation just over a decade ago, alarm bells were sounded about the possibility of its addition to soils resulting in an increased loss of nSOC and increased CO_2_ emissions (8–10). These observations ignited a flurry of research into the potential interactions between added PyC and nSOC. This research was important because if PyOM additions are to be used for climate change mitigation, the added PyC must not be offset by increased nSOC losses. Initial investigations revealed a range of responses, spanning from large increases in nSOC mineralization to large decreases in SOC mineralization with PyOM additions (11–13). (Although the term “priming” [14] is widely used to describe this phenomenon, due to broad interpretations of the term [15], we refer to “increased or decreased mineralization”; even though less concise, this method- and process-agnostic term will help ensure clarity and avoid prior expectations of what the term priming implies.) Research over the past decade has progressed beyond the observation of the phenomenon to systematic investigations of the mechanisms underlying these interactions (16–18), while the conclusions from meta-analyses have strengthened as the total number of studies of PyOM-SOC interactions has steadily increased (19–22).

The above-cited meta-analyses provide a robust overview of recent advances in the literature. Briefly, the current understanding of mechanisms underlying the interactive effects of PyOM additions on SOC mineralization includes the following observations (19–22). (i) In general, when changes in mineralization occur, net increases in nSOC mineralization tend to be limited to the earlier stages of incubations or field studies, while net decreases in nSOC mineralization often emerge later. (ii) It is essential to consider the specific properties of PyOM and the soil to which it is applied in tandem. Properties such as pH, total nSOC content, nutrient status, and texture or particle size are important determining factors of the net C effects of PyOM additions on nSOC. (iii) Specific researcher-determined conditions of the study can significantly affect the response variables of interest. This is particularly true for moisture and duration of the experiment. Although the above-mentioned factors make it challenging to collectively develop a predictive understanding of interactions between SOC and PyOM mineralization, it is important to design experiments explicitly to test for and quantify the relative importance of specific mechanisms. In this spirit, in this study, we sought to investigate short-term increases in SOC mineralization with PyOM amendments.

Although numerous studies have now observed net decreases in SOC mineralization with PyOM amendments over the long term, characterization of the mechanisms that underpin both of these phenomena will help us develop appropriate models for predicting long-term effects into the future (23, 24). For example, in a C cycling model designed to predict the long-term effects of PyOM on C stocks (23), the assumption is that the dominant mechanism of decreased SOC mineralization is sorption of SOC by the PyOM, which is represented in the model by decreasing the fraction of SOC that is partitioned to the more rapidly cycling pool. However, the assumption for increased SOC mineralization is that the dominant mechanism is increased microbial activity, which is represented in the model by increasing the rate at which nSOC is mineralized. These assumptions create a model structure that helps drive the model’s predictions of long-term net decreases in nSOC mineralization with PyOM additions. Although increases in nSOC mineralization rates after PyOM additions seem to be limited to short and medium (<2-year) timelines (21, 22), we wanted to investigate these short-term effects since they pose the greatest risk of unintended consequences for nSOC stocks during intentional PyOM additions as biochar for C management or for increased nSOC losses due to PyOM inputs after wildfires.

Commonly proposed mechanisms for short-term increases in nSOC mineralization with PyOM additions can be broadly grouped into two categories: (i) cometabolism, where easily mineralizable PyOM fractions increase microbial activity, resulting in the additional decomposition of SOC, and (ii) stimulation, where PyOM additions may result in changes to the soil chemical or physical environment that generally favor increased microbial activity, such as more optimal pH, nutrient, oxygen, or water conditions (19, 20). In addition, community composition shifts could also help explain these phenomena (25). It is possible that PyOM additions could induce changes to the microbial community composition that shift the community toward taxa that favor different sources of organic matter or process organic matter differently, e.g., organisms with different carbon use efficiencies (CUEs) (18). Finally, researchers often distinguish these effects from “apparent priming,” when total CO_2_ emissions from soil increase but this increase is not accompanied by increases in nSOC losses (15). Rather, the increase is attributed to an increased turnover of soil microbial biomass. While the effects included under stimulation are essential to understand in order to predict SOC fluxes, they are, mechanistically, comparably straightforward: researchers have long studied the effects of changing moisture or oxygen on SOC fluxes. If we are able to quantify the degree to which PyOM additions to soil change these properties, we will be on our way to predicting their effects on nSOC cycling. However, the effects included under cometabolism and community composition shifts are generally less well characterized, and it is these mechanisms that we specifically sought to investigate in this study.

While research into the mechanisms behind changes in SOC mineralization with PyOM additions has grown substantially over the last decade, our understanding of which microbes respond to PyOM additions, and the reasons for their response, has somewhat lagged behind, particularly for fungi. As an exception to this, a recent investigation by Yu et al. into the effects of PyOM on SOC mineralization included an assessment of bacteria and fungi using high-throughput sequencing, through which they identified that the relative abundances of the fungal classes *Sordariomycetes* and *Tremellomycetes* were significantly positively correlated with increases in SOC mineralization after 40 days of incubation (26). In our recent review of PyOM effects on soil bacterial communities (27), we reanalyzed papers published before 2018 that had publicly accessible data and used Illumina high-throughput sequencing of the 16S rRNA gene to characterize soil bacterial communities (25, 28–32). Using the same approach to reanalyze all data sets, we found the following: (i) although most communities were significantly altered by the addition of PyOM, rather than creating a “charosphere”-dominated community (33, 34), PyOM-amended soil bacterial communities resembled their corresponding unamended soil communities more closely than they resembled different soils that had also been amended with PyOM; (ii) phylum-level responses to PyOM additions were not consistent across different soil and PyOM combinations (i.e., the taxonomic level is generally too broad to make meaningful conclusions about soil bacterial responses to PyOM); and (iii) a small number of taxa were identified as being PyOM responders in more than one study, most of which came from the classes *Actinobacteria*, *Alphaproteobacteria*, and *Betaproteobacteria* (27). Based on these findings, we suggest that the field is still too nascent to make broad generalizations about any kind of consistent effect of PyOM on microbial communities and hope that continuing to blend functional measurements with microbial response data will help to identify which specific microbes might be responsible for changes in nSOC mineralization with PyOM additions while also generally increasing our understanding of which microbes respond to PyOM additions and why.

In this study, we had two research questions, with alternate hypotheses for each. Our first question was, are soils with less SOC more prone to stimulation by PyOM additions? Our primary hypothesis was that soils with less nSOC are more likely to experience increased mineralization with PyOM additions via cometabolism. Our alternate hypothesis rationalized the opposite: soils with less nSOC may be less likely to experience increased short-term mineralization with PyOM additions. This could occur if the microbial communities were limited by mineral nutrients. If PyOM additions alleviated this constraint via stimulation, microbial communities in soils with more mineralizable OC might be better able to take advantage of this subsidy. Our second question was, do soil microbial communities reflect changes in nSOC mineralization with PyOM additions? Our primary hypothesis was that there would be larger changes to the microbial community (larger Bray-Curtis dissimilarities between amended and unamended soils) in the soils where PyOM additions increased nSOC mineralization, while microbial communities in soils that did not experience increased nSOC mineralization would not change as much (smaller Bray-Curtis dissimilarities between amended and unamended soils). The rationale was that groups of microbes that respond positively to PyOM additions may be the same groups that are responsible for increased nSOC mineralization with PyOM additions, so a stronger shift toward these groups may accompany a stronger effect on nSOC mineralization. Our first alternate hypothesis was that PyOM might change microbial communities similarly in all soils: if PyOM additions had a very strong effect on the microbial community composition, creating a consistent charosphere community, dissimilarities between amended and unamended soils across soil types might not be statistically significant. Our second alternate hypothesis was that we might not see significantly different communities at all with PyOM additions. Although previous studies have seen significant changes to microbial communities with PyOM additions (27), these studies have often added extremely large amounts of PyOM. When applied at environmentally relevant rates, while PyOM additions may provide an additional C source, it may be relatively small in comparison to the available nSOC, and any effects of PyOM additions on the soil water-holding capacity, pH, or nutrient availability may not be large enough to significantly alter the soil microbial community composition from the unamended soil communities.

## RESULTS

We incubated five contrasting soils with a range of SOC stocks from sites across the United States (Table 1) that had previously been stored at −80°C, adding ^13^C-labeled corn stover (“OM”), PyOM produced at 350°C from the same corn stover (“PyOM”), or no additions (“soil”). We monitored CO_2_ fluxes over 4 weeks and used stable isotope partitioning to separate CO_2_ emissions into SOC- and amendment-derived pools. We characterized the microbial (bacterial/archaeal and fungal) communities after 24 h, 10 days, and 26 days using rRNA gene sequencing (16S and internal transcribed spacer 2 [ITS2]) as they rebounded from freezing storage and responded to amendments.

**TABLE 1:** Studied soils and their properties

Source location	Soil type	C concn (%)	N concn (%)	Ca concn (mg kg^−1^)	Mg concn (mg kg^−1^)	Na concn (mg kg^−1^)	K concn(mg kg^−1^)	pH
Laupahoehoe, HI	Typic Hydrudand	33.1	2.0	492	137	29	86	5.0
Caribou Creek-Poker Flats, AK	Pergelic Cryaquept	9.4	0.4	540	68	16	20	5.0
Ithaca, NY	Typic Fragiudept	4.6	0.4	1,219	202	87	119	5.1
Onaqui, UT	Xeric Haplocalcid	2.6	0.1	5,619	245	28	511	8.3
Ordway-Swisher Biological Station, FL	Lamellic Quartzipsamment	0.5	0.02	77	14	11	8	5.2

**FIG 1:**
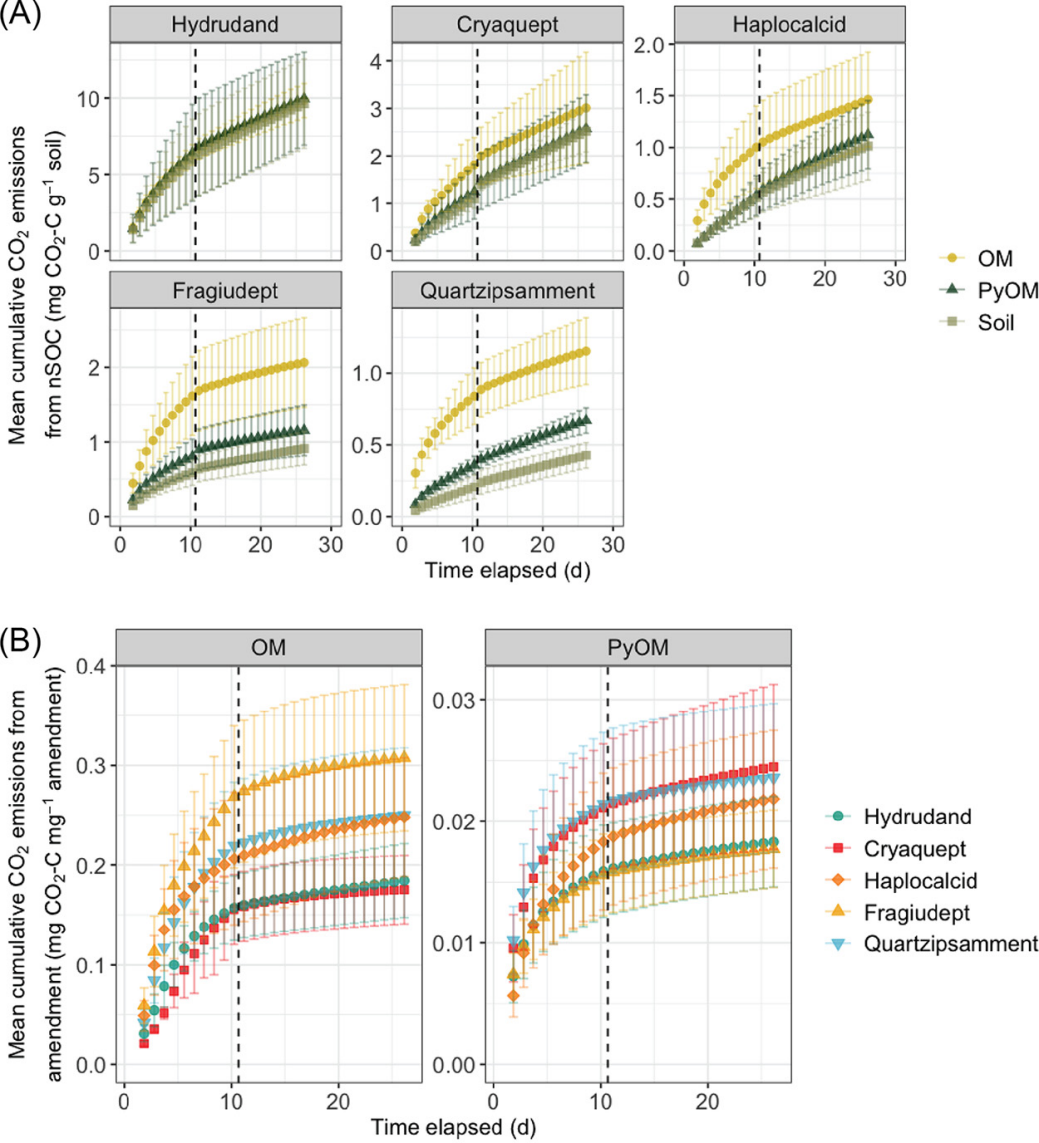
(A) Mean cumulative nSOC-derived CO_2_ emissions over time for each soil, with organic matter (OM) additions, pyrogenic organic matter (PyOM) additions, or no additions (Soil). Error bars represent ±1.96 standard errors (SE) (95% confidence intervals). The dashed lines indicate the sampling point for midincubation harvests (*n* = 4). Note the different scales on the *y* axes. (B) Mean cumulative amendment-derived CO_2_ emissions over time. Error bars represent ±1.96 SE (95% confidence intervals). The dashed lines indicate the sampling points for midincubation harvests (*n* = 4). Note the different scales on the *y* axes.

**FIG 2:**
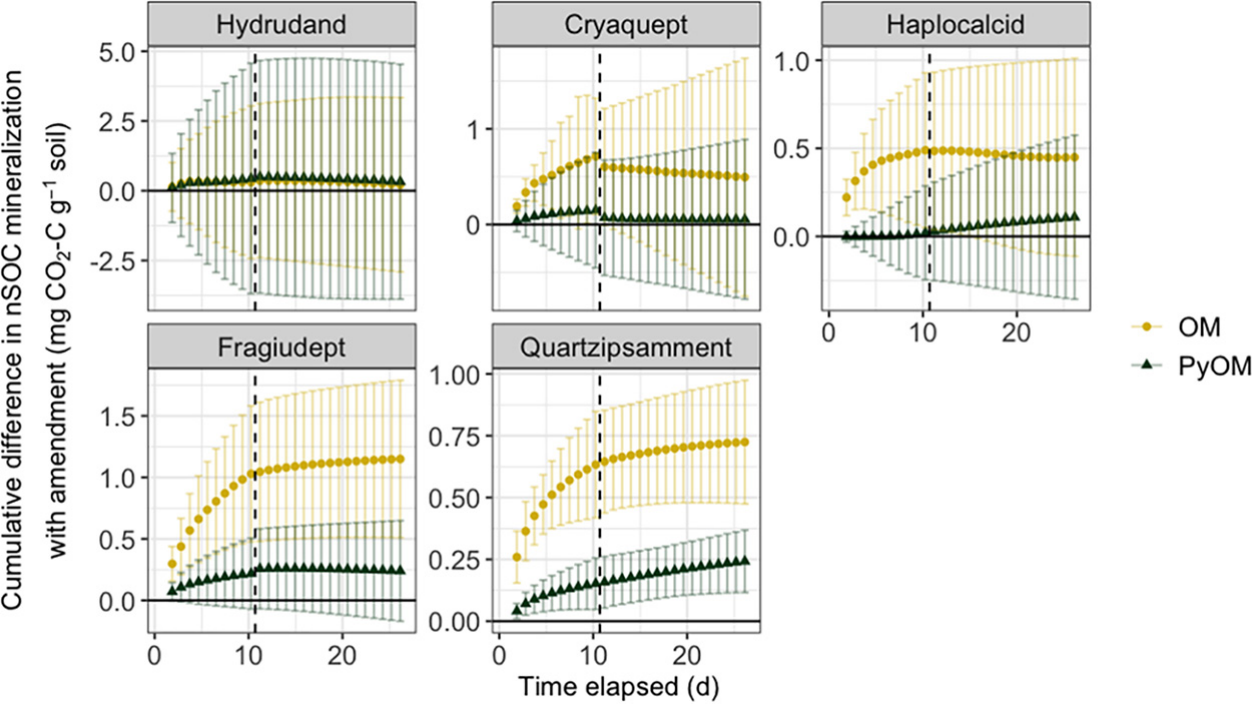
Mean cumulative difference in nSOC-derived CO_2_ emissions in amended soils compared to unamended soil over time for each soil, with organic matter (OM) additions and pyrogenic organic matter (PyOM) additions. Error bars represent ±1.96 SE (95% confidence intervals), where nonoverlapping bars indicate significant differences. The dashed lines indicate the sampling points for midincubation harvests (*n* = 4). Note the different scales on the *y* axes.

nSOC-derived CO_2_ emissions were highest in the soils with the most total SOC (the Hydrudand and Cryaquept) and lowest in the soils with less total SOC (the Haplocalcid, Fragiudept, and Quartzipsamment) (Fig. 1). PyOM additions increased cumulative nSOC-derived CO_2_ emissions by 55% for the Quartzipsamment soil (Florida) only, while OM additions increased cumulative nSOC-derived CO_2_ emissions by 44% for the Haplocalcid (Utah), by 126% for the Fragiudept (New York), and by 170% for the Quartzipsamment (Florida) soils (Fig. 1 and 2). These effects were generally largest earlier in the incubation periods, although the significant effects persisted throughout the full 26 days for the Quartzipsamment.

**FIG 3:**
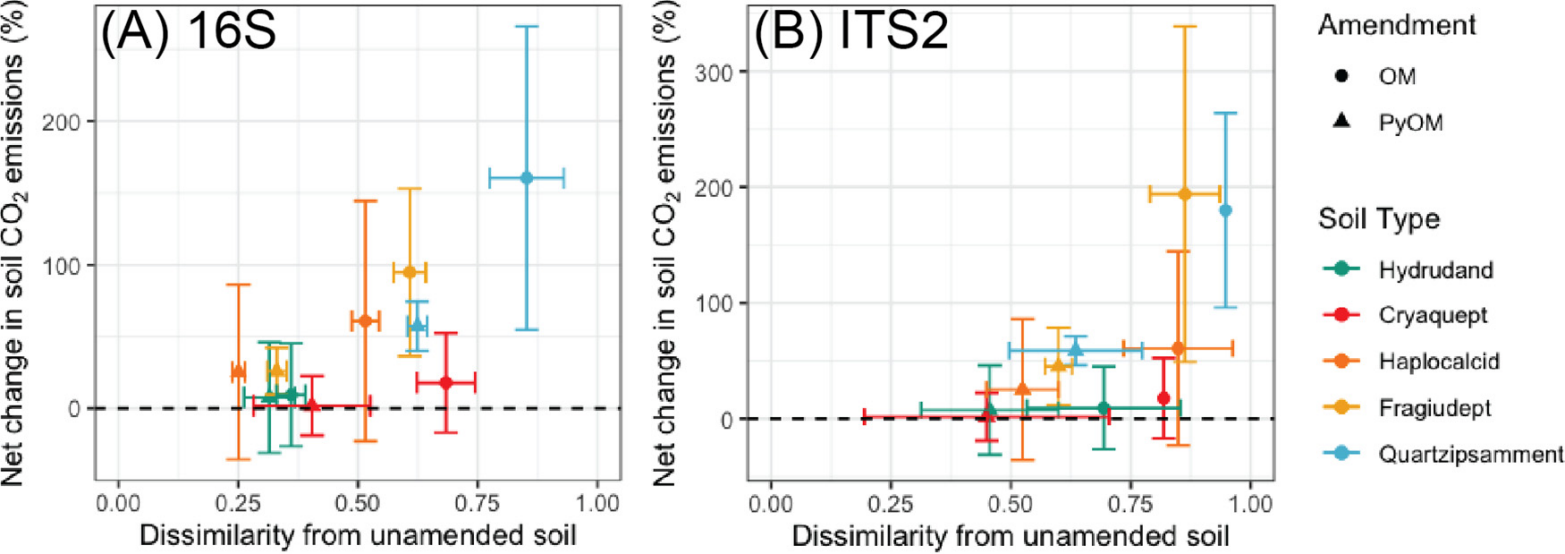
Net change in soil-derived CO_2_ emissions (percent) versus Bray-Curtis dissimilarity on Hellinger-transformed abundances from unamended soil for different previously frozen soils and amendments at the final time point (day 26). Error bars represent ±1.96 SE (95% confidence intervals). (A) *Bacteria* and *Archaea* (16S) (*n* = 4, except for Quartzipsamment, where *n* = 3). (B) *Fungi* (ITS2) (*n* = 4).

**FIG 4:**
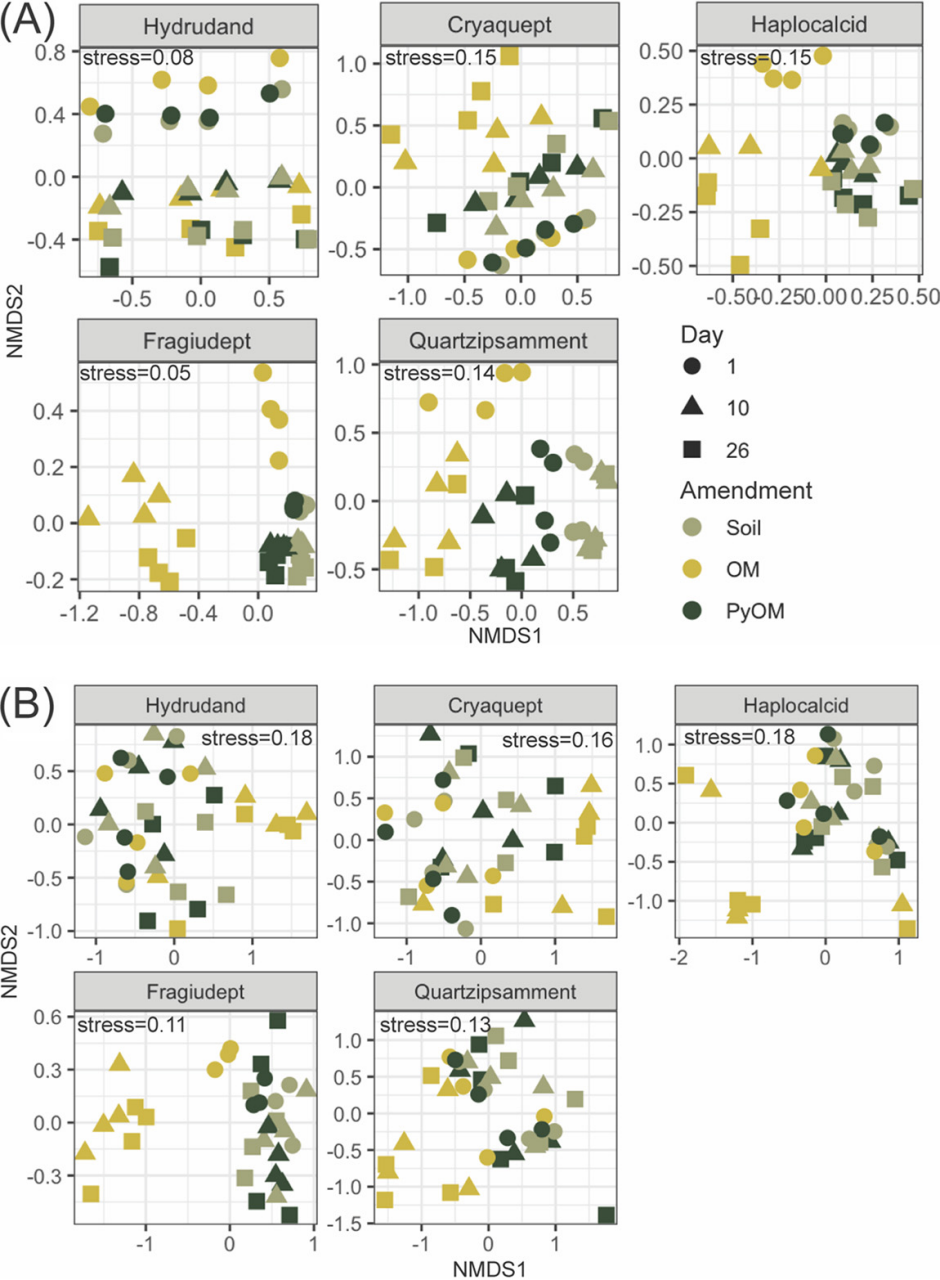
Nonmetric multidimensional scaling plots of Bray-Curtis dissimilarities between soil microbial communities (Hellinger-transformed relative abundances) at all three time points (1 day, 10 days, and 26 days) for each (previously frozen) soil. Shapes indicate whether organic matter (OM), pyrogenic organic matter (PyOM), or nothing (soil) was added. (A) *Bacteria* and *Archaea* (16S) (*k* = 2, stress_Hydrudand_ = 0.08, stress_Cryaquept_ = 0.15, stress_Haplocalcid_ = 0.15, stress_Fragiudept_ = 0.05, and stress_Quartzipsamment_ = 0.14) (*n* = 4 for each time point, except for Haplocalcid on day 10 and Quartzipsamment on day 26, where *n* = 3). Ordinations were performed individually for each soil type. (B) Fungi (ITS2) (*k* = 2, stress_Hydrudand_ = 0.18, stress_Cryaquept_ = 0.16, stress_Haplocalcid_ = 0.18, stress_Fragiudept_ = 0.11, and stress_Quartzipsamment_ = 0.13) (*n* = 4 for each time point, except for Fragiudept on day 1, where *n* = 3). Ordinations were performed individually for each soil type.

The greatest changes in soil community composition (highest Bray-Curtis dissimilarity from unamended soil) upon amendment with PyOM or OM were associated with the greatest increases in nSOC-derived CO_2_ emissions (Fig. 3). For the full data set, bacterial and fungal community compositions were both significantly affected by soil type, days of incubation, amendment, and interactions between soil type and day and between soil type and amendment (*P* < 0.001 for all effects by permutational multivariate analysis of variance [PERMANOVA]) (see Tables S4 and S5 and Fig. S1 and S2 in the supplemental material). Although our goal was not to determine the underlying causes of differences between communities in the unamended soils, all tested soil properties, pH, cation exchange capacity (CEC), Ca, Mg, Na, K, total C, and total N, were significantly correlated (*P* < 0.05 by PERMANOVA) with community composition for the 16S and the ITS2 data sets (Tables S6 and S7). For bacteria and archaea, when the soils were analyzed individually (Fig. 4A), days of incubation and amendment were both significant predictors of bacterial community composition (*P* < 0.02 by PERMANOVA), except for the Hydrudand, where only days of incubation were significant (Table S5). The effects of amendments on bacterial community composition were least pronounced in the Hydrudand from Hawaii and the Cryaquept from Alaska and most pronounced for the Quartzipsamment from Florida (Table S8). For the fungi, when the soils were analyzed individually (Fig. 4B), amendment was a significant predictor of fungal community composition for all soils except the Cryaquept (*P* < 0.007 by PERMANOVA), and days of incubation were significant for the Hydrudand, Cryaquept, and Fragiudept (*P* < 0.03 by PERMANOVA) (Table S9). The effects of amendments on fungal community composition were most pronounced in the Fragiudept from New York (Table S9).

**FIG 5:**
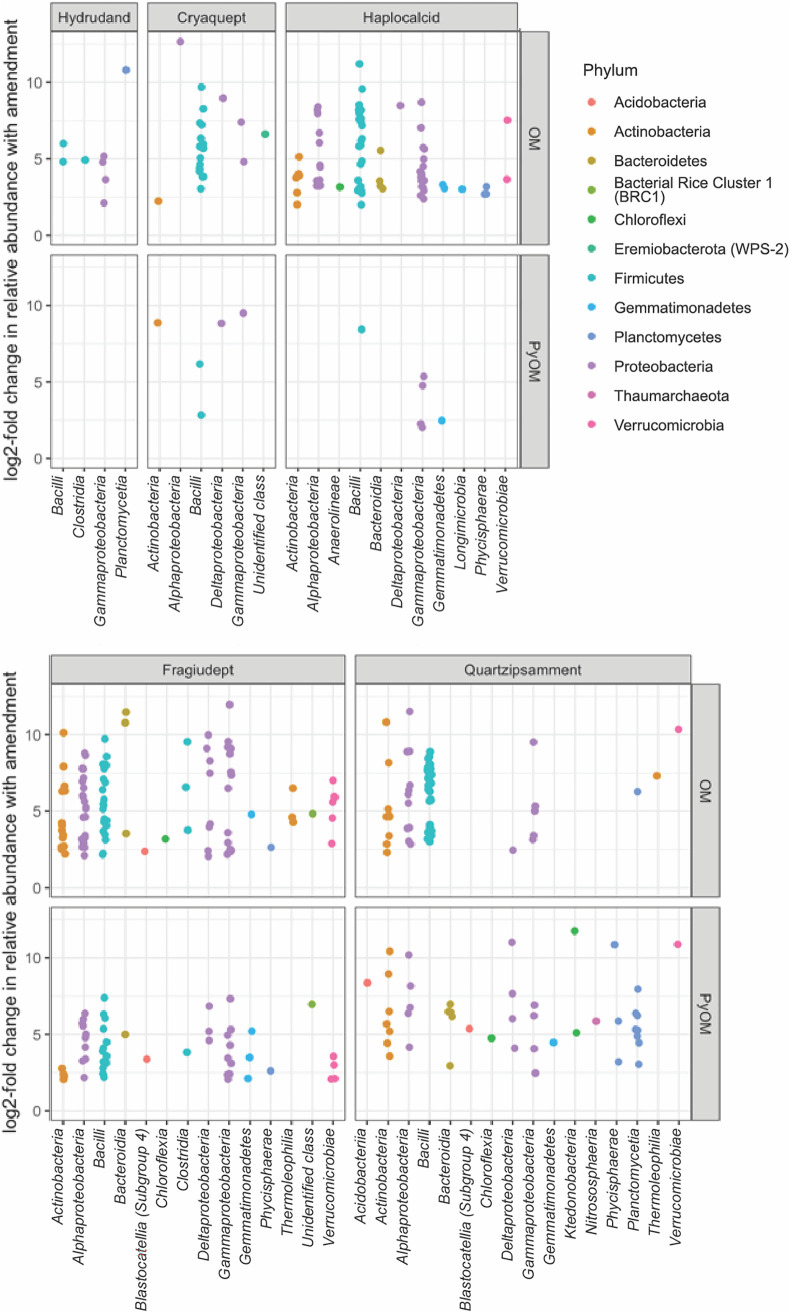
Differential abundances of bacterial and archaeal OTUs that are positive responders (log_2_ fold change of ≥2) to OM or PyOM additions in previously frozen soils, as estimated using the “corncob” algorithm (35) and grouped by soil and class. Each point represents a single OTU.

**FIG 6:**
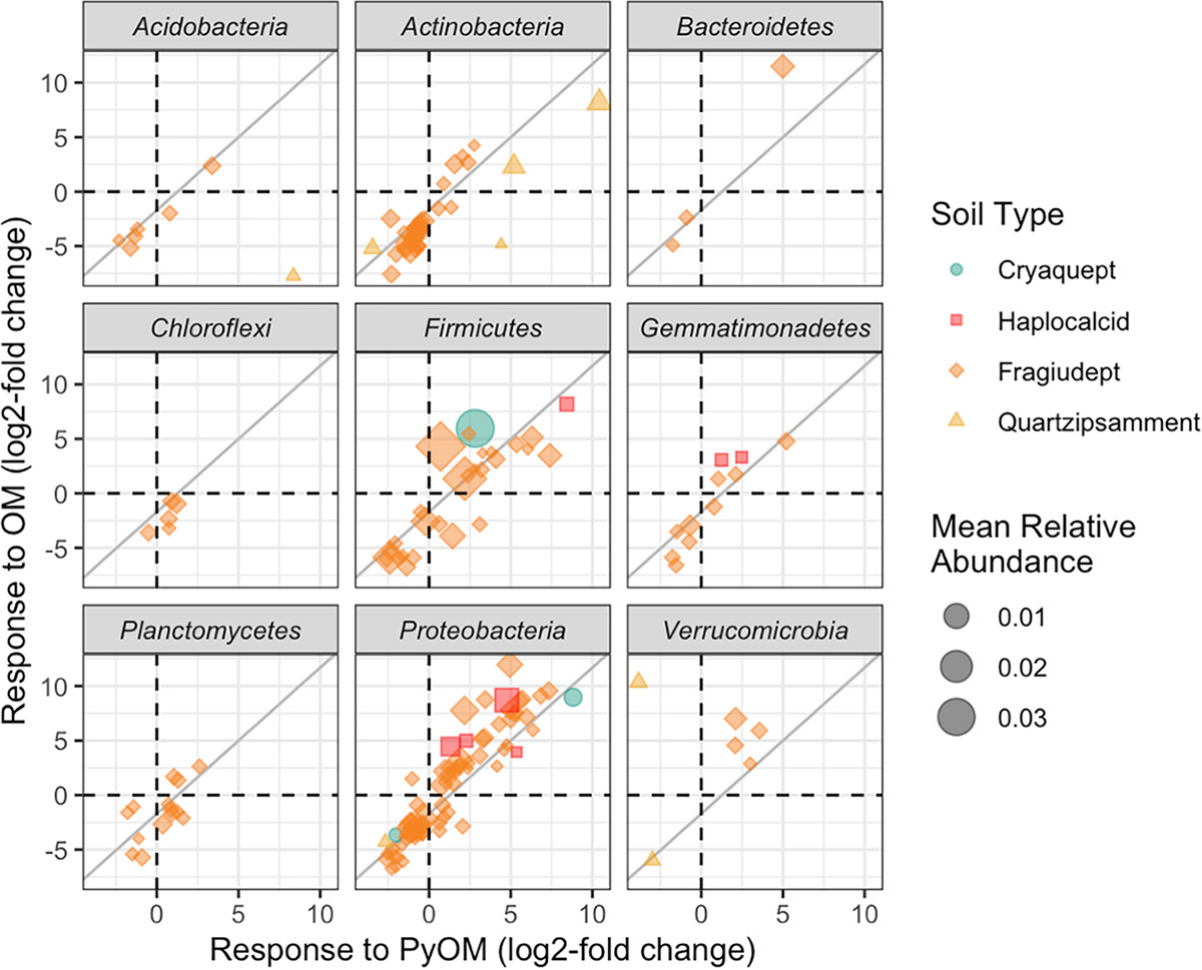
Response to PyOM versus response to OM for bacterial OTUs that were present at a mean of at least 0.01% and that were present in OM-amended, PyOM-amended, and unamended previously frozen soils, as estimated using the corncob algorithm (35). Each point represents a single OTU from one soil, with color and shape indicating soil source and size scaled by mean relative abundance within a soil, across all treatments, on days 10 and 26. Dashed black lines indicate zero, or no change in relative abundance compared to unamended soil. The solid gray lines indicate the linear fit for all phyla [(response to OM) = −1.7 + 1.3 × (response to PyOM); *P* < 0.001; *R*^2^_adj_ = 0.63].

Across all soils, we identified 258 16S operational taxonomic units (OTUs) that responded positively to OM amendments and 162 16S OTUs that responded positively to PyOM amendments (Fig. 5; Table S10). Only three ITS2 OTUs, *Spizellomyces* in the Fragiudept and *Penicillium* and *Sporormiaceae* in the Haplocalcid, were identified as being significant positive responders to PyOM, and no fungi were positive responders to OM over the time frame of this study (Table S11). Of the responsive 16S OTUs, 77 were responders to PyOM in at least one soil as well as to OM in at least one soil, or “common positive responders.” Genera with common positive responders in multiple soils included *Flavisolibacter* (3 OM-responsive OTUs in 1 soil and 3 PyOM-responsive OTUs across 2 soils), *Bacillus* (29 OM-responsive OTUs across all 5 soils and 6 PyOM-responsive OTUs across 2 soils), *Microvirga* (18 OM-responsive OTUs across 3 soils and 8 PyOM-responsive OTUs across 3 soils), and *Noviherbaspirillum* (8 OM-responsive OTUs across 3 soils and 5 PyOM-responsive OTUs across 2 soils). With a few exceptions, bacterial taxa that responded positively or negatively to PyOM tended to also respond similarly to OM (Fig. 6). There was a significant positive relationship between the responses to the two amendments (*P* < 0.0001 in a linear model).

## DISCUSSION

### Effects of organic amendments on nSOC-derived CO_2_ reflect baseline soil C status.

In response to our first question, our findings were consistent with our primary hypothesis: soils with lower baseline CO_2_ emissions experienced greater increases in nSOC mineralization with additions of OM or PyOM (Fig. 1 and 2). Simultaneously, increases in nSOC mineralization were greater with additions of OM than with additions of PyOM. These results are consistent with the idea that the activities of such microbial communities are more likely to be limited by C availability such that the addition of PyOM could alleviate this constraint, resulting in generally increased microbial activity and, thus, increased SOC mineralization. In particular, the already low-C Quartzipsamment from Florida was especially vulnerable to increased nSOC losses with amendments. Although the Haplocalcid and Fragiudept soils also tended to have increased nSOC losses with the addition of PyOM, the Quartzipsamment was the only soil for which this effect was statistically significant for PyOM additions. These findings are consistent with previous studies across a range of soils and SOC contents (19–22). However, it is important to note that numerous other mechanisms could also contribute meaningfully to increased nSOC mineralization with organic amendments, as observed in other systems (17, 19, 20) and as described in the introduction. However, we do not believe that the effects that we observed were primarily driven by pH shifts: the pHs of four of the five soils were very similar (5.0 to 5.2). Additionally, we do not believe that the increases in nSOC mineralization were driven primarily by the effects of the amendments on moisture: we adjusted moisture individually for each treatment. We do not believe that the effects were driven primarily by alleviation of a nutrient constraint with the addition of PyOM: the PyOM had relatively low N, and furthermore, previous studies have often shown that soil CO_2_ emissions are inhibited by mineral N additions (36). Additionally, although the strongly responding Quartzipsamment had the lowest measured mineral nutrient concentrations (Ca, Mg, and K) (Table 1), the highest/second-highest-nutrient soil was the Fragiudept, and it had the next strongest CO_2_ response to PyOM and OM amendments, suggesting that alleviation of nutrient limitations with PyOM or OM additions was not the dominant mechanism driving our observed effects.

On the one hand, the fact that the amendments had the least effect on the high-C soils suggests that, overall, the effects of increased nSOC mineralization with PyOM amendments might be less concerning since the highest-C soils are less responsive. On the other hand, one might interpret it as being more concerning since soils with the lowest SOC and the lowest microbial activity to begin with are most at risk for increased nSOC losses with PyOM amendments. This raises the question of which soils would be the best candidates for OM or PyOM additions. High-C soils seem to have lower risks for short-term increased CO_2_ emissions. However, other benefits to low-C soils, such as changes to the water-holding capacity or total SOC content (PyOM-C plus SOC), might outweigh this trade-off.

Even though our results strongly support the finding that short-term increases in CO_2_ emissions are most likely to occur in soils with low C and/or low mineralization rates to begin with, it is important to note that these effects were observed over the short term, i.e., over just a few weeks. As in other studies, in the time period during which amendments increased net nSOC-derived CO_2_ emissions, the net increase usually began to level off or even began to decrease. Given this observation, and since other studies have observed net negative effects of PyOM amendments on nSOC-derived CO_2_ emissions over longer time periods (16, 17), the findings from this study should be considered primarily within the context of short-term responses to amendments.

### The magnitude of microbial community composition change mirrors the magnitude of increases in nSOC-CO_2_.

In response to our second question, our findings were also consistent with our primary hypothesis: we found that the degree to which soil microbial communities change with PyOM or OM amendments reflected the degree to which nSOC mineralization also increased (Fig. 4). From these results, we hypothesize that the taxa that respond positively to PyOM and especially OM additions may also be the same taxa that are responsible for increased nSOC mineralization with PyOM or OM additions. Thus, a stronger shift toward these groups is accompanied by a stronger effect on nSOC mineralization. That said, it is important to note that because we did not directly trace the fate of the organic substances into taxon-specific microbial biomass (e.g., using an approach such as stable isotope probing), we have not conclusively demonstrated that the microbes that increased in abundance with additions were also the ones that metabolized the larger amount of nSOC; rather, this would be a future hypothesis to test. Overall, OM additions resulted in both a larger change in community composition and a larger increase in nSOC mineralization than did PyOM additions.

### PyOM responders differ across soils and do not reflect a common charosphere.

Although PyOM additions had a significant effect on microbial community composition, PyOM-induced changes in community composition were much smaller than the differences in community composition between different soils (Fig. 3; see also Tables S4 and S5 and Fig. S1 and S2 in the supplemental material). Thus, PyOM did not result in a community dominated by the “charosphere” (33, 34) but, rather, resulted in detectable but relatively subtle shifts within a few of the existing taxa (Fig. 5 and 6). We made a similar observation in our recent review of the effects of PyOM additions on soil bacterial community composition (27). The current study substantially improves our confidence in that observation since it is not constrained by the challenges of cross-study differences in methods and materials and spans five different soils. Together, these observations underscore the importance of considering the effects of PyOM within the unique context of a given soil rather than generalizing the effects of PyOM on soil microbial communities across all soils.

We were also interested in the specific taxa that responded to PyOM additions. In a previous field trial with the same Fragiudept soil and similar amendments (25), we identified a number of “common responders” to PyOM and OM after 82 days in the field. We suggested that those taxa may most likely be responsible for the short-term C mineralization effects of PyOM additions, and we predicted that we would observe a similar phenomenon in the current study, possibly even across soils. This general trend persisted (Fig. 6) in that the OTU responses to the two amendments were significantly positively correlated (*P* < 0.0001); i.e., taxa that responded (positively or negatively) to one amendment tended to respond similarly to the other. Although there are a few taxa that are exceptions to this (they respond positively to one amendment but negatively to the other), we hesitate to dwell too much on this response since they tend to be low-abundance taxa to begin with. Because the same taxa that responded to PyOM over the short term also responded positively to OM, we suggest that this supports the idea that PyOM-responsive taxa in this study were likely responding to the small fraction of easily mineralizable PyOM-C, supporting the idea that a responsive fraction of the overall community might be responsible for short-term increases in nSOC mineralization with PyOM amendments. Over longer timescales, we might expect different results as other mechanisms emerge. However, we were not necessarily able to identify a “core set” of PyOM responders across different soils. This is likely due in part to the small response overall to PyOM in the higher-C soils and also to the diversity of organisms between soils. While there were 162 different PyOM-responsive OTUs, the same OTUs were often not present in the different soils: 62% of all 16S OTUs were detected (regardless of abundance) in only a single unamended soil (97% for ITS2), and 26% of all 16S OTUs were detected in only two different soils (2% for ITS2). In particular, since we used the dada2 OTU-picking algorithm, which can differentiate OTUs that differ by a single base pair, or “amplicon sequence variants,” it may be useful to consider common responders at a coarser phylogenetic scale. If we consider the OTUs at the genus level, there were numerous bacterial genera with OTUs that were responsive to PyOM in multiple soils, as well as OM amendments, as described in Results. Some of the genera with PyOM-responsive OTUs across more than one soil were also identified as having PyOM-responsive OTUs in multiple studies in our previous meta-analysis, including *Flavisolibacter*, *Microvirga*, and *Noviherbaspirillum* (27). Additionally, some of these PyOM-responsive bacteria are from genera that have been identified as being fire responsive in other studies (e.g., *Microvirga* [37], *Bacillus* [38], and *Noviherbaspirillum* [39]), although we remind the reader that taxa even from the same genus can differ meaningfully in their functions (40). Because all of the named taxa were also responsive to OM amendments over the short term, we raise the question of whether these OTUs may be responding to the more easily mineralizable fractions of PyOM or, in the case of fires, also to fire-released OM. Together, these taxa could represent interesting candidates for future investigation of the ecology of fire- and PyOM-responsive bacteria.

### Conclusions and outlook.

While our short-term incubation indicates that low-C soils might be at the greatest risk for short-term C losses with OM or PyOM amendments, we note that the losses were greater with OM than with PyOM additions and that many studies have shown that these short-term effects are relatively limited and often even become net C increases over longer timescales. Together, our findings indicate that changes in microbial community composition mirrored changes in nSOC mineralization. This suggests that it may be likely that the change in CO_2_ emissions with the addition of amendments is governed by a specific subset of the microbial community, rather than a general stimulation of the entire community. Although these specific responsive organisms were not consistent across all soils and depend on the native microbial community, certain taxa were identified as common responders. Future research could utilize techniques such as stable isotope probing to conclusively demonstrate which microbes are using the amendments as a C source and to expand the research to more soil types, different timescales, and different PyOM materials to begin to develop a more comprehensive understanding of the specific microbial responders. It would also be interesting to determine whether or when our observation does not hold: whether there are conditions under which large community changes in response to organic amendments are not accompanied by changes in nSOC-CO_2_ emissions and, conversely, whether there are conditions where large changes in CO_2_ emissions are observed but not accompanied by changes in microbial community composition.

## MATERIALS AND METHODS

### Soil descriptions.

Soil properties are described in Table 1. Four of the soils were collected from National Ecological Observatory Network (NEON) sites according to NEON protocols in 2009 to 2010, as part of a NEON prototype study, from the top 0 to 0.1 m of the A horizon (41). We added a local non-NEON site in which we had previously investigated PyOM effects on SOC cycling and microbial communities (the Fragiudept/New York site). Each sample replicate was from a single core, except for the Cryaquept and Fragiudept, which were from composited cores. Samples were stored at −80°C until experimental initiation, except for being shipped overnight to Ithaca, NY, on dry ice. Clearly, this treatment would be expected to have an effect on the specific soil community composition after thawing and incubation (42). Due to logistical constraints in collecting fresh soils from all sites, and in order to treat all soils equivalently, we worked with frozen samples. Thus, we would expect our findings for these soils with respect to factors such as the identification of specific taxa as having the ability to respond to PyOM amendments or the positive relationship between total microbial community composition shifts and increased nSOC mineralization with amendments to remain applicable in other systems, while the specific numeric values (e.g., baseline abundances of individual taxa or absolute magnitude of CO_2_ fluxes) and taxa that were not identified as positive responders should not be directly translated to natural ecosystems.

### Corn stover and PyOM amendment production.

^13^C pulse-labeled corn [Zea mays (L.)] shoot biomass was grown, ground (<2 mm), and pyrolyzed at 350°C under Ar gas in a modified muffle furnace as previously described in detail (43). Amendment properties are reported in Table S1 in the supplemental material.

### Incubation setup and monitoring.

Frozen samples were thawed, sieved through a <2-mm sieve, and air dried at room temperature until the mass stabilized with losses changing by less than 1% per day. A subsample was rapidly dried at 70°C in a drying oven and used to determine the moisture-holding capacity individually for each soil, with each amendment, in order to ensure that all samples are at equivalent moisture levels given that amendments might affect the water-holding capacity. To do this, we weighed the soil samples (amended or unamended) in a PVC tube with a screen covered by a moist filter paper at the bottom. The tubes were placed into a container, and water was slowly added to the container until the samples were saturated and the level of the water was level to the surface of the soil. The saturated soils were allowed to stand overnight. In the morning, they were removed from the water bath and allowed to drain freely overnight, covered in parafilm. The mass of water remaining in the soil was taken to represent the “field capacity” (FC), with a target moisture value for incubation of 65% FC. We also calculated the final moisture content of the air-dried soil to enable us to calculate the water required to reach this value for the incubations.

We prepared separate incubation vials for each treatment to be sampled at each time point. For vials with amendments, we added OM at 3% by mass, and added PyOM on a prepyrolysis mass basis, which resulted in a 0.99% by mass addition; i.e., we added the mass of PyOM that would have remained if we used the same amount of initial biomass to produce PyOM, essentially asking the systems-level question, what might the fate of this biomass be? Based on our expectations for CO_2_ flux rates from previous experiments, we determined that we would require 1 g soil per incubation for the high-organic-matter soils (typic Hydrudand and perigelic Cryaquept) and 5 g per incubation for the lower-organic-matter soils in order to make sure that CO_2_ fluxes remained within the optimal range for our instrumentation setup. For the 24-h time points, we used 2 g of soil to conserve soil. Each jar, amended and unamended, was stirred to mix. The experiment was initiated (time zero [*t*_0_]) for each jar when water was added to bring it up to 65% FC. At wet-up, each jar received water dropwise to gradually bring it up to the target moisture level. The vial for the 24-h time point was incubated at 30°C for 24 h in Mason jars with 20 ml deionized water (DIW) in the bottom to maintain a moist environment and was then destructively sampled for microbial community composition after exactly 24 h by collecting the entire sample in a Whirl-Pak bag. The sample was immediately frozen at −80°C and stored until DNA extraction, except for overnight shipment on dry ice to Madison, WI. The two vials for the two later time points, 10 days and 26 days, were placed into the same quart-size Mason jar along with 20 ml DIW in the bottom of the Mason jar to maintain a moist environment. The Mason jar was then sealed with a lid with tubing connected to the gas monitoring system. Because each full measurement cycle on the gas monitoring system takes 20 min, one experimental treatment was wet up every 20 min, taking care to attach it to the gas monitoring system at the corresponding time. The jars were automatically sampled using a custom-built multiplexer system (see reference 17 for details) connected to a cavity ring-down spectrometer (G2201-I; Picarro, Santa Clara, CA, USA) that measures CO_2_ concentrations and ^13^CO_2_/^12^CO_2_ isotopes. Measurements were made on a continuous monitoring cycle, which resulted in each Mason jar being measured about once a day. After 10 days, jars were opened, and one vial was randomly removed to be destructively sampled for microbial community characterization. Mason jars were removed and returned on a time cycle to ensure that each vial was sampled at the equivalent time since wet-up. After 26 days, the second vial was removed and destructively sampled for microbial community characterization.

### DNA extraction and sequencing.

DNA extractions were performed for each sample and for the original materials (OM and PyOM), with one blank extraction for every 24 samples (identical methods but using empty tubes, all of which were sequenced). We used a DNeasy PowerLyzer PowerSoil DNA extraction kit (Qiagen, Germantown, MD) according to the manufacturer’s instructions and bead-beating samples for 45 s at 6 m s^−1^ on a FastPrep 5G homogenizer (MP Biomedicals, Santa Ana, CA). Extracted DNA was amplified in triplicate PCRs, targeting the 16S rRNA gene v4 region (here “16S”) with primers 515f (GTGYCAGCMGCCGCGGTAA) and 806r (GGACTACNVGGGTWTCTAAT) (44) and targeting the ITS2 gene region with primers 5.8S-Fun (AACTTTYRRCAAYGGATCWCT) and ITS4-Fun (AGCCTCCGCTTATTGATATGCTTAART) (45), with barcodes and Illumina sequencing adapters added as described previously (46) (Table 2; see Tables S3 and S4 in the supplemental material for full primers with all barcodes and adapters). The PCR amplicon triplicates were pooled, purified, and normalized using a SequalPrep normalization plate (96) kit (Thermo Fisher Scientific, Waltham, MA). Samples, including blanks, were pooled, and library cleanup was performed using a Wizard SV gel and PCR cleanup system (catalog number A9282; Promega, Madison, WI). The pooled library was submitted to the University of Wisconsin—Madison Biotechnology Center (Madison, WI) for 2-by-250 paired-end (PE) Illumina MiSeq sequencing for the 16S amplicons and 2-by-300 PE sequencing for the ITS2 amplicons.

**TABLE 2:** PCR and sequencing primers used in this study^*a*^

Primer	Sequence^*b*^
16S Illumina	
515f	AATGATACGGCGACCACCGAGATCTACAC*XXXXXXXX***TATGGTAATTGT**GTGYCAGCMGCCGCGGTAA
806r	CAAGCAGAAGACGGCATACGAGAT*XXXXXXXX***AGTCAGCCAGCC**GGACTACNVGGGTWTCTAAT
Read 1 seq	TATGGTAATTGTGTGYCAGCMGCCGCGGTAA
Read 2 seq	AGTCAGCCAGCCGGACTACNVGGGTWTCTAAT
Barcode seq	ATTAGAWACCCBNGTAGTCCGGCTGGCTGACT

ITS2 Illumina	
ITS4	AATGATACGGCGACCACCGAGATCTACAC*XXXXXXXX***TATGGTAATTAA**AGCCTCCGCTTATTGATATGCTTAART
5.8S	CAAGCAGAAGACGGCATACGAGAT*XXXXXXXX***AGTCAGTCAGGG**AACTTTYRRCAAYGGATCWCT
Read 1 seq	TATGGTAATTAAAGCCTCCGCTTATTGATATGCTTAART
Read 2 seq	AGTCAGTCAGGGAACTTTYRRCAAYGGATCWCT
Barcode seq	AGWGATCCRTTGYYRAAAGTTCCCTGACTGACT

a See references 44–46. Full Illumina PCR primers with barcodes are listed in Tables S2 and S3 in the supplemental material.

b The barcode is shown in italic type, and the pad and linker are shown in boldface type.

### Microbial community bioinformatics.

For 16S reads (32,000 minimum, 208,000 maximum, and 58,000 median total sequenced reads), we quality filtered and trimmed, dereplicated and learned errors, assigned operational taxonomic units (OTUs), and removed chimeras using dada2 (47) as implemented in R (mean of 53% of the initial reads remaining after the full pipeline; 15,000 minimum, 180,000 maximum, and 29,000 median total final reads). Taxonomy was assigned to the 16S reads using a naive Bayes classifier (48) trained on the 515f-806r region of the 99% identity OTUs from the Silva nr 132 database (49, 60) as implemented in QIIME2 (50). We removed any OTUs classified as chloroplasts or mitochondria. For ITS2 reads (21,000 minimum, 289,000 maximum, and 62,000 median total sequenced reads), we first merged reads using PEAR (51) and then performed the same steps as described above for 16S (mean of 50% of the initial reads remaining after the full pipeline; 6,000 minimum, 199,000 maximum, and 32,000 median total final reads). Taxonomy was assigned to the ITS2 reads using the UNITE general release dynamic threshold database (2 February 2019) using a naive Bayes classifier (48) as implemented in dada2 (47). We removed any OTUs that did not receive a classification at the phylum level in order to exclude any nonfungal ITS2 sequences. High-memory-intensive sequence processing steps were performed at the UW-Madison Center for High Throughput Computing cluster (Madison, WI).

### Stable isotope CO_2_ flux partitioning.

Respiration data were analyzed as described previously (17) using R version 3.6.1 (52). Sample respiration was partitioned between the amendment-derived CO_2_-C and soil-derived CO_2_-C using the equations δ_measured_ = δ_soil_ × *f*_soil_ + δ_amendment_ × *f*_amendment_ and CO_2_-C_total_ = CO_2_-C_soil_ + CO_2_-C_amendment_, where δ represents the δ^13^C signature (with respect to the PeeDee belemnite standard) of the total respired CO_2_-C (δ_measured_), the soil-derived CO_2_ (δ_soil_), or the amendment-derived CO_2_-C (δ_amendment_) and *f* represents the fraction of the total CO_2_-C derived from the soil (*f*_soil_) or the amendment (*f*_amendment_) (53). δ^13^C of bulk PyOM (δ_PyOM_) or bulk OM (δ_OM_) was used as the amendment end-member for isotope partitioning. Soil isotope end-members (δ_soil_) to be used in isotope partitioning were obtained daily using the average δ^13^C for CO_2_-C from control (unamended) treatments (see the R scripts at https://github.com/TheaWhitman/NEON_PyOM). We interpret values that do not overlap in a 95% confidence interval as being significantly different.

### Microbial community analyses.

We worked primarily in Jupyter notebooks, with phyloseq (54), ggplot (55), and dplyr (56) being instrumental in working with the data in R (52). We compared community compositions across samples using Bray-Curtis dissimilarities (57) on Hellinger-transformed relative abundances (58), which we represented using nonmetric multidimensional scaling (NMDS) ordinations. We tested for significant effects of soil site, days of incubation, amendment, and interactions between soil and day and between soil and amendment using permutational multivariate analysis of variance (PERMANOVA) (the adonis function in vegan [59]). We also tested whether baseline soil properties—pH, CEC, Ca, Mg, Na, K, total C, and total N—were correlated with the initial community composition in the unamended soils using PERMANOVA. We identified OTUs that were differentially abundant (significantly enriched in amended soils compared to control soils) within each soil type and amendment, testing only taxa that represented at least 0.01% of the mean total community for that soil using the R package corncob (35). We analyzed the two later time points together, while controlling for time point and controlling for differential variance, using a Wald test and correcting *P* values to yield a false discovery rate of less than 0.05 within each soil type and amendment. We fit a linear model for the response to OM versus the response to PyOM for taxa with significant responses to at least one of the amendments.

### Data availability.

Sequencing data are available in the NCBI SRA under BioProject accession number PRJNA687484 and BioSample accession numbers SAMN17145005 to SAMN17145200. Code used to analyze data and generate figures in this paper is available at https://github.com/TheaWhitman/NEON_PyOM.

## Supplementary Material

Supplemental file 1

Supplemental file 2

Supplemental file 3

Supplemental file 4

Supplemental file 5
