# Supplementary material for: Microbial Community Shifts Reflect Losses of Native Soil Carbon with Pyrogenic and Fresh Organic Matter Additions and Are Greatest in Low-Carbon Soils
Source: Appl Environ Microbiol. 2021 Mar 26;87(8):e02555-20. doi: 10.1128/AEM.02555-20 (PMC8091118; doi:10.1128/AEM.02555-20)
Supplement: Supplemental file 1 [file AEM.02555-20-s0001.pdf]

Supplemental Information for:

Microbial community shifts reflect losses of native soil carbon with pyrogenic and fresh organic matter additions and are greatest in low-carbon soils

Thea Whitman[a,b*], Silene DeCiucies[b], Kelly Hanley[b], Akio Enders[b], Jamie Woolet[a], and Johannes Lehmann[bc]

a. Department of Soil Science, University of Wisconsin, Madison, WI 53706, USA
b. Soil and Crop Sciences, School of Integrative Plant Science, Cornell University, Ithaca, NY 14850, USA
c. Atkinson Center for a Sustainable Future, Cornell University, Ithaca, NY 149850, USA
* Corresponding author at 1525 Observatory Dr., Madison, WI, 53706, USA; email: twhitman@wisc.edu

Supplemental Table S1. Initial PyOM and corn stover properties

**Property (units)**	**Value**	
	PyOM	Stover
Total C (%)	58.5	41.3
Total N (%)	2.7	1.96
C:N (by mass)	22	21
C isotope signature ($\delta^{13}C$, ‰)	+711.4	+741.9
Total H (%)	3.9	
Total O (%)	15	
pH_{DIW} (1:20 w/v)	10.0	
Feedstock	Corn	
Particle size (mm)	< 2	
Heating rate (°C min^{-1})	5	
Final temp (°C)	350	
Residence time (min)	45	
Surface area (m^2 g^{-1})	92.8	
ASTM Ash (%)	17	
ASTM Volatiles (%)	35	
ASTM Fixed C (%)	48	

Supplemental Table S2. 16S Illumina PCR primers (available as .csv)

Supplemental Table S3. ITS2 Illumina PCR primers (available as .csv)

Supplemental Table S4. PERMANOVA on mixed model 16S Bray-Curtis dissimilarities on Hellinger-transformed OTU table

	**Df**	**SumsOfSqs**	**MeanSqs**	**F.Model**	**R^2**	**Pr(>F)**
**Soil Name**	4	35.255	8.8136	68.374	0.54022	0.001*
**Amendment**	2	1.732	0.8662	6.72	0.02655	0.001*
**Day**	2	1.433	0.7163	5.557	0.02195	0.001*
**Soil Name x Amendment**	8	4.223	0.5279	4.095	0.06471	0.001*
**Soil Name x Day**	8	3.411	0.4264	3.308	0.05227	0.001*
Residuals	149	19.207	0.1289		0.29431	

Supplemental Table S5. PERMANOVA on mixed model ITS2 Bray-Curtis dissimilarities on Hellinger-transformed OTU table

	**Df**	**SumsOfSqs**	**MeanSqs**	**F.Model**	**R^2**	**Pr(>F)**
**Soil Name**	4	25.121	6.2802	22.7928	0.31247	0.001*
**Amendment**	2	1.892	0.9461	3.4338	0.02354	0.001*
**Day**	2	1.361	0.6805	2.4697	0.01693	0.001*
**Soil Name x Amendment**	8	6.125	0.7656	2.7785	0.07618	0.001*
**Soil Name x Day**	8	4.016	0.5019	1.8217	0.04995	0.001*
Residuals	152	41.881	0.2755		0.52094	

Supplemental Table S6. PERMANOVA on mixed model 16S Bray-Curtis dissimilarities on Hellinger-transformed OTU table for unamended initial soils

**Parameter**	**Df**	**SumsOfSqs**	**MeanSqs**	**F.Model**	**R^2**	**Pr(>F)**
pH	1	1.7385	1.73852	16.0955	0.25249	0.001
CEC	1	0.6621	0.66214	6.1302	0.09617	0.001
Ca	1	1.2517	1.25173	11.5888	0.1818	0.001
Mg	1	0.5573	0.55728	5.1594	0.08094	0.001
Na	1	0.4067	0.40671	3.7654	0.05907	0.001
K	1	0.2131	0.21314	1.9732	0.03095	0.036
Total C	1	0.2534	0.25341	2.3461	0.0368	0.014
Total N	1	0.6143	0.61431	5.6874	0.08922	0.001
Residuals	11	1.1881	0.10801	0.17256		
Total	19	6.8854	1			

Supplemental Table S7. PERMANOVA on mixed model ITS2 Bray-Curtis dissimilarities on Hellinger-transformed OTU table for unamended initial soils. Parameters are listed in the order in which they were entered into the model.

**Parameter**	**Df**	**SumsOfSqs**	**MeanSqs**	**F.Model**	**R^2**	**Pr(>F)**
pH	1	1.035	1.03498	3.7379	0.12493	0.001
CEC	1	0.7169	0.71687	2.589	0.08653	0.001
Ca	1	1.0324	1.03237	3.7284	0.12461	0.001
Mg	1	0.696	0.69599	2.5136	0.08401	0.001
Na	1	0.5537	0.55373	1.9998	0.06684	0.001
K	1	0.4211	0.42105	1.5206	0.05082	0.026
Total C	1	0.4525	0.45249	1.6342	0.05462	0.017
Total N	1	0.6082	0.60816	2.1964	0.07341	0.001
Residuals	10	2.7689	0.27689	0.33422		
Total	18	8.2845	1			

Supplemental Table S8. PERMANOVA on mixed model 16S Bray-Curtis dissimilarities on Hellinger-transformed OTU tables for each soil type

	**Factor**	**Df**	**SumOfSqs**	**MeanSqs**	**F.Model**	**R^2**	**Pr(>F)**
Hydrudand	Amendment	2	0.211	0.10552	0.6428	0.03127	0.877
	**Day**	2	1.4483	0.72414	4.4113	0.21462	0.001*
	Residuals	31	5.0888	0.16416	0.75411		
	Total	35	6.7482	1			
Cryaquept	**Amendment**	2	0.9431	0.47156	2.7581	0.12437	0.001*
	**Day**	2	1.3399	0.66995	3.9185	0.1767	0.001*
	Residuals	31	5.3	0.17097	0.69893		
	Total	35	7.5831	1			
Haplocalcid	**Amendment**	2	0.8438	0.42188	5.4826	0.23716	0.001*
	**Day**	2	0.5595	0.27977	3.6358	0.15727	0.001*
	Residuals	28	2.1545	0.07695	0.60558		
	Total	32	3.5578	1			
Fragiudept	**Amendment**	2	1.4381	0.71907	11.9733	0.36461	0.001*
	**Day**	2	0.6444	0.32221	5.3653	0.16338	0.001*
	Residuals	31	1.8617	0.06006	0.47201		
	Total	35	3.9443	1			
Quartzip-samment	**Amendment**	2	2.5193	1.25965	7.3456	0.30827	0.001*
	**Day**	2	0.8514	0.42571	2.4825	0.10418	0.001*
	Residuals	28	4.8016	0.17148	0.58754		
	Total	32	8.1723	1			

Supplemental Table S9. PERMANOVA on mixed model ITS2 Bray-Curtis dissimilarities on Hellinger-transformed OTU tables for each soil type

	**Factor**	**Df**	**SumOfSqs**	**MeanSqs**	**F.Model**	**R^2**	**Pr(>F)**
Hydrudand	**Amendment**	2	1.0546	0.52732	2.0764	0.10152	0.007*
	**Day**	2	1.4615	0.73073	2.8774	0.14068	0.004*
	Residuals	31	7.8726	0.25396	0.75781		
	Total	35	10.3887	1			
Cryaquept	Amendment	2	1.0224	0.51119	1.6602	0.08671	0.059
	**Day**	2	1.2232	0.61161	1.9863	0.10374	0.031*
	Residuals	31	9.5454	0.30791	0.80955		
	Total	35	11.791	1			
Haplocalcid	**Amendment**	2	1.3992	0.69958	2.3483	0.12354	0.001*
	Day	2	0.6914	0.34569	1.1604	0.06105	0.28
	Residuals	31	9.2351	0.29791	0.81542		
	Total	35	11.3257	1			
Fragiudept	**Amendment**	2	3.018	1.50898	10.8799	0.37986	0.001*
	**Day**	2	1.0435	0.52176	3.7619	0.13134	0.001*
	Residuals	28	3.8835	0.1387	0.4888		
	Total	32	7.945	1			
Quartzip-samment	**Amendment**	2	1.5226	0.76131	2.0803	0.11014	0.004*
	Day	2	0.957	0.47848	1.3075	0.06922	0.136
	Residuals	31	11.3447	0.36596	0.82064		
	Total	35	13.8243	1			

Supplemental Table S10. 16S responders to amendments across soils

Supplemental Table S11. ITS2 responders to amendments across soils

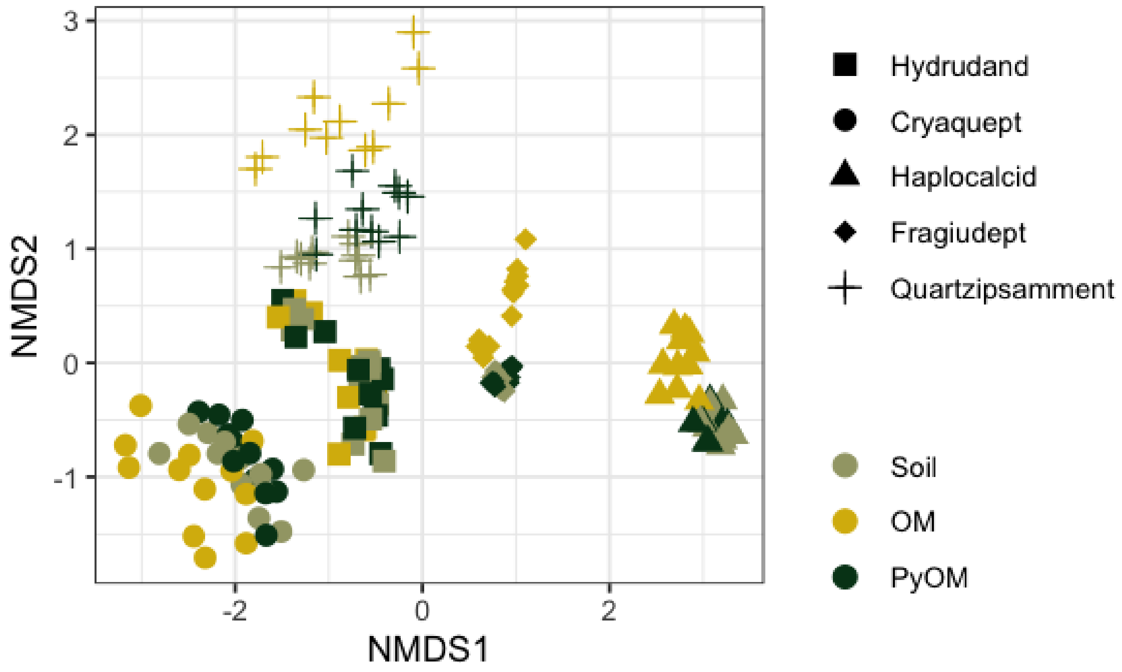

Supplemental Figure S1. Non-metric multidimensional scaling plot of Bray-Curtis distances between soil bacterial and archaeal communities (Hellinger-transformed relative abundances) at all three timepoints (not distinguished on figure) for each soil. Colors indicate whether organic matter (OM, yellow), pyrogenic organic matter (PyOM, dark green), or nothing was added (Soil, light green) and shapes indicate soil site. k=2, stress=0.10.

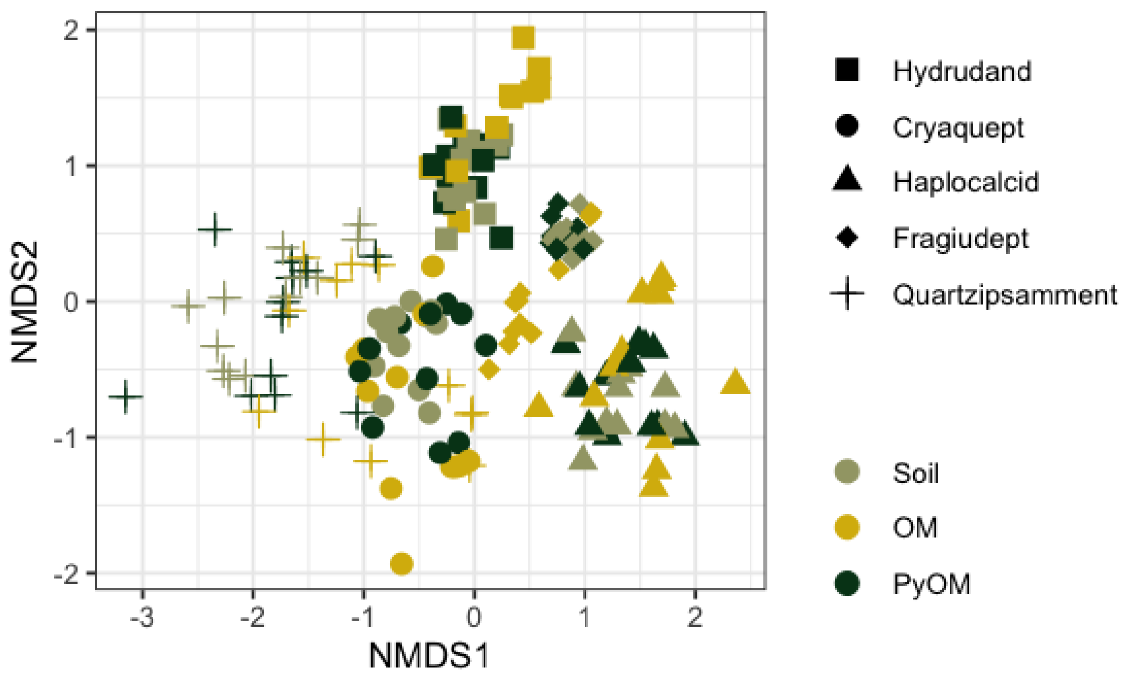

Supplemental Figure S2. Non-metric multidimensional scaling plot of Bray-Curtis distances between soil fungal communities (Hellinger-transformed relative abundances) at all three timepoints (not distinguished on figure) for each soil. Colors indicate whether organic matter (OM, yellow), pyrogenic organic matter (PyOM, dark green), or nothing was added (Soil, light green) and shapes indicate soil site. k=2, stress=0.10.
